# Towards a Multi-omics Understanding of Anaphylaxis: Insights into Pathogenesis and Biomarker Identification

**DOI:** 10.1007/s12016-025-09069-8

**Published:** 2025-06-30

**Authors:** Manca Svetina, Tanja Kunej, Peter Korošec, Matija Rijavec

**Affiliations:** 1 University Clinic of Respiratory and Allergic Diseases Golnik, Golnik, Slovenia; 2 Biotechnical Faculty, University of Ljubljana, Ljubljana, Slovenia; 3 Faculty of Pharmacy, University of Ljubljana, Ljubljana, Slovenia

**Keywords:** Anaphylaxis, Multi-omics, Pathophysiology, Biomarker, Precision medicine

## Abstract

Anaphylaxis is a severe, life-threatening hypersensitivity reaction that presents significant challenges in both clinical practice and scientific research. While individual omics studies have provided valuable insights into the genetic predisposition, immune dysregulation, and metabolic alterations associated with anaphylaxis, a comprehensive understanding of its full pathophysiology remains elusive. Multi-omics integration, which combines genomics, epigenomics, transcriptomics, proteomics, and metabolomics, has the potential to uncover novel mechanisms, biomarkers, and therapeutic targets. However, studies employing comprehensive multi-omics approaches in anaphylaxis are still limited. This review of 107 studies published between 2000 and 2024—including genomics (43), metagenomics (2), epigenomics (2), transcriptomics (20), proteomics (26), and metabolomics (14)—synthesizes findings from existing single-omics studies on human anaphylaxis, identifies key interconnections across omics layers, and underscores the critical need for large-scale, integrative research. Advancing this type of research is essential to advance our understanding of anaphylaxis, improve risk prediction, and enhance both diagnosis and treatment strategies.

## Introduction

Anaphylaxis is a life-threatening systemic hypersensitivity reaction that poses significant challenges to both clinicians and researchers [1]. Despite its acute onset and severity, the molecular mechanisms driving anaphylaxis remain incompletely understood. This knowledge gap hinders the development of effective therapeutic strategies and reliable biomarkers for early diagnosis and prognosis [1]. Although the development of anaphylaxis has been known to be influenced by both genetic and environmental factors, the exact mechanisms driving its onset and severity remain unclear. The IgE-mediated reaction —where high-affinity IgE receptor (FcεRI)-bound IgE on mast cells and basophils is cross-linked by an antigen—is considered a central mechanism in the anaphylaxis pathogenesis [1]. However, emerging evidence suggests that non-IgE-mediated pathways, such as the activation of complement receptors C3a/C5a or the Mas-related G protein-coupled receptor-X2 (MRGPRX2), also play significant roles in this process [2]. Upon activation, mast cells release a diverse array of mediators crucial to the inflammatory response observed during anaphylaxis [1]. Furthermore, ongoing research highlights the involvement of additional effector cells, signalling pathways, and mediators that may contribute to the full spectrum of anaphylactic symptoms [1]. The condition exhibits significant heterogeneity, with patients displaying diverse clinical manifestations [3] and varying responses to treatment [4, 5]. Identifying novel biomarkers to predict the risk of a severe reaction, confirm the diagnosis of anaphylaxis, and distinguish specific mechanisms and pathophysiology remains a major challenge. Furthermore, without better-defined clinical endotypes, personalized treatment approaches and accurate risk prediction remain difficult.[1, 6, 7].

Recent advancements in high-throughput omic technologies—including genomics, transcriptomics, epigenomics, proteomics, and metabolomics—offer promising opportunities to construct detailed molecular profiles and enhance our understanding of anaphylaxis [8, 9]. The complexity of diseases like anaphylaxis cannot be fully understood through single-omics methodologies, as they fail to capture the interactions between complex molecular pathways. Multi-omics could provide a more comprehensive perspective on the disease, enabling the identification of specific biomarkers and the discovery of potential therapeutic targets [9, 10].

Despite significant advancements in omics sciences, comprehensive reviews addressing multi-omics approaches in anaphylaxis remain scarce. This review systematically analyzes studies utilizing omics techniques in anaphylaxis. We aimed to integrate findings from diverse omics studies to provide a broader and more detailed understanding of its underlying mechanisms.

## Materials and methods

### Screening Strategy and Selection Criteria

This systematic review was conducted using a structured and comprehensive methodology to identify relevant studies. Publications were retrieved from PubMed using the keyword “anaphylaxis” in combination with one of the following terms: “chromatin”, “DNA”, “epigenetics”, “epigenome”, “epigenomics”, “exposome”, “exposomics”, “expression”, “gene”, “genome”, “genomics”, “GWAS”, “histone”, “lncRNA”, “metabolomics”, “metagenomics”, “methylation”, “microarray”, “microbiome”, “miRNA”, “mRNA”, “mutation”, “ncRNA”, “omic”, “polymorphism”, “protein”, “proteome”, “proteomics”, “RNA”, “SNP”, “transcript”, “transcriptomics”, “variant”, “mediator”. The terms were searched for articles published between January 1, 2000, and December 31, 2024. This comprehensive approach aimed to capture a wide range of relevant studies. Duplicate entries, non-English articles, and studies that did not include human samples were excluded. Two reviewers independently assessed each study for inclusion. Any disagreements were resolved through discussion. No automation tools were used in the process. Subsequently, the data were categorized based on omics methodologies. To classify the omics approaches, we adapted the hierarchy according to the proposed taxonomy for multi-omics sciences [11]. Gene names, sequence variants, and chromosomal locations were verified using the Ensembl Genome Browser (https://www.ensembl.org/index.html) [12], ClinVar (https://www.ncbi.nlm.nih.gov/clinvar/) [13] and the HGNC database (https://www.genenames.org/) [14]. DNA, RNA, and protein sequence variants were described according to the HGVS nomenclature [15]. Protein terminology was standardized using the UniProt database (https://www.uniprot.org/) [16].

## Results and Discussion

**Fig. 1:**
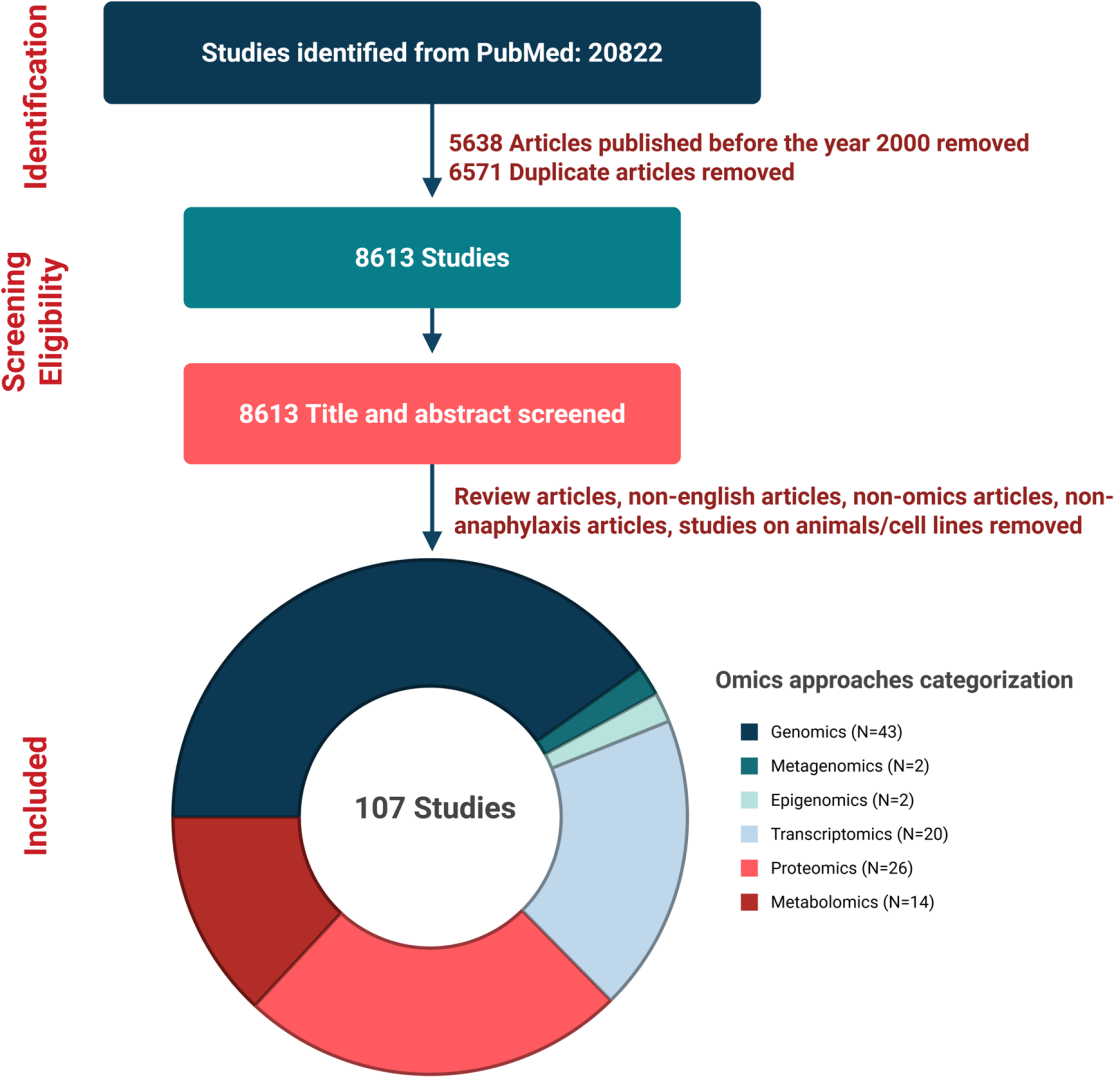
Flow diagram of the screening process and the number of different omics approach studies included in the review

PubMed screening identified 20,822 articles, with 15,184 articles published between the year 2000 and the end of 2024. After the removal of duplicate records, 8613 anaphylaxis-related articles remained. A systematic screening process resulted in the selection of 107 studies for detailed manual review and omics analysis. These included 43 studies in genomics, 2 in metagenomics, 2 in epigenomics, 20 in transcriptomics, 26 in proteomics, and 14 in metabolomics (Fig. 1). Several studies included in the review were not omics-based; however, they were retained due to their relevance.

### Genomics and Metagenomics

The advent of genomics has expanded genetics, enabling the analysis of complete gene sets (genomes) and providing deeper insights into genetic systems [17]. However, conducting large-scale genome-wide association studies (GWAS) on anaphylaxis remains challenging because anaphylaxis is a rare and often unpredictable event, making it difficult to obtain sufficiently large case cohorts for statistically robust analyses. Our literature screening identified only four genome-wide studies [18–21]. Two studies on immediate allergic reactions to β-lactams found strong associations in the *HLA-DRA*|*HLA-DRB5* interregion and *HLA-DRB1*[18, 19]. Another study investigated wheat-dependent exercise-induced anaphylaxis (WDEIA) and identified the HLA-DPB1*02:01:02 allele as a major genetic risk factor [20]. *HLA* region was also identified as a surrogate genetic marker for perioperative anaphylaxis in a whole-exome association study [22]. Additionally, HLA-DRB1*15:02, HLA-DRB*12:01, HLA-DRB1*04:03, and HLA-DRB1*14:54 were associated with anaphylaxis induced by iodinated contrast media and drugs [23–25], further confirming the role of *HLA* variants in immediate hypersensitivity. The fourth study, investigating anaphylaxis after diclofenac etalhyaluronate injection, found no clear associations, likely due to a small sample size [21].

Most genetic studies on anaphylaxis have focused on individual genes rather than the entire genome. Candidate gene studies, which examine specific genes based on prior knowledge of immune pathways, are more common due to their feasibility and targeted approach. Since mast cells play a central role in anaphylaxis, genetic susceptibility factors affecting their activation and mediator release have been extensively studied. The p.D816V activating variant in the tyrosine kinase KIT (*KIT* p.D816V), which promotes mast cell differentiation and proliferation, was first reported in 2005 as being more prevalent in individuals with anaphylaxis compared to the general population [26]. Subsequent studies have consistently confirmed the significance of the *KIT* p.D816V variant in the development of anaphylaxis [26–32]. These studies demonstrated that the variant is not only more prevalent in patients experiencing anaphylaxis but also contributes to the severity of reactions, especially in *Hymenoptera* venom-triggered anaphylaxis (HVA) [30, 31, 33]. The presence of the *KIT* p.D816V variant is strongly associated with clonal mast cell disorders, including systemic mastocytosis and monoclonal mast cell activation syndrome, which predispose individuals to heightened anaphylactic risk [34]. Consequently, screening for the *KIT* p.D816V variant has gained prominence as an important diagnostic tool, particularly for identifying individuals at high risk of severe reactions and those with underlying clonal disorders, thereby guiding treatment strategies for these patients [32, 34, 35]. Besides *KIT* variants, copy number variations (CNV) in the *TPSAB1* gene encoding α-tryptase have emerged as important contributors to severe anaphylaxis [30, 32, 34, 36–42]. Hereditary alpha-tryptasemia (HαT) is an autosomal dominant condition caused by increased copy numbers of *TPSAB1* encoding α-tryptase [43]. Unlike *KIT* p.D816V, a somatic variant in clonal mast cell disorders, HαT is a germline trait linked to elevated basal tryptase and heightened mast cell activation through α/β-tryptase tetramers [44]. HαT is relatively common, present in 5–7% of Western populations, and is not inherently disease-causing, as individuals with HαT can be asymptomatic [45].

T helper-type 2 cytokines, such as interleukin-4 (IL-4) and IL-13, may play a central role in allergic inflammation [46]. The protein known as signal transducer and activator of transcription 6 (STAT6) is a key transcription factor involved in both IL-4- and IL-13-mediated biological responses [46, 47]. Multiple studies have identified *STAT6* variants, including gain-of-function, associated with anaphylaxis [46–51]. STAT3, another key transcription factor in immune regulation, is primarily associated with autosomal dominant hyper-IgE syndrome (AD-HIES), a condition characterized by eczema, recurrent infections, and high IgE levels. Although anaphylaxis is rare in AD-HIES, a documented case suggests that *STAT3* variants may contribute to severe allergic responses in certain individuals [52].

Filaggrin, encoded by the *FLG* gene, is a protein essential for maintaining skin barrier integrity, and loss-of-function variants in this gene are associated with food allergen sensitization [53]. Two studies involving families with WDEIA and children with food-induced anaphylaxis have indicated that *FLG* variants may contribute to the pathogenesis of anaphylaxis [54, 55].

The remaining genetic studies on anaphylaxis identified variations in inflammatory and immune-related genes (*NLRP3*[56], *IL4*[57], *IL10*[58], *IL18*[59]), metabolic enzyme genes (*ACE* [60], *AGT* [61, 62], *NAT2* [63]) and other genes like *HP* [64] and *CEP68* [65] as potentially important, though each was reported in only one study (Table 1).

**Table 1:** Sequence variants in genetic regions associated with anaphylaxis

Pathology	Gene symbol	Gene name	Sequence variant*	Genome location (GRCh38)	Traditional name	Reference
WDEIA	***HLA-DPB1***	Major histocompatibility complex, class II, DP beta 1	HLA-DPB1*02:01:02			[20]^♦^
Anaphylaxis to drugs	**Intergenic, intron variants**		rs117659975, rs118159062, rs2339299, rs1877655, rs2531853	chr6:66163499G>T; chr14:104112091A>G; chr15:33696903G>A; chr2:110955126C>T; chr17:9299937T>G		[21]^♦^
Anaphylaxis to drugs/contrast media	***HLA-DRB1***	Major histocompatibility complex, class II, DR beta 1	HLA-DRB1*15:02			[23]
			HLA-DRB1*04:03, HLA-DRB1*14:54			[25]
Anaphylaxis to drugs	***HLA-G***	Major histocompatibility complex, class I, G	NM_001384280.1:c.366C>T; p.(His122 =); rs1130356	chr6:29828550C>T		[22]
Anaphylaxis to drugs	***LIMD1***	LIM Domain Containing 1	NM_014240.3:c.1409-5G>T, p.?; rs62242177 NM_014240.3:c.1409-4C>G, p.?; rs62242178	chr3:45636145G>T; chr3:45636146C>G		[25]
Anaphylaxis to insect venom/food/drugs/idiopathic	***KIT***	KIT proto-oncogene, receptor tyrosine kinase	NM_000222.3:c.2447A>T, p.(Asp816Val)	chr4:54733155A>T	p.D816V	[26] [27] [28] [29] [31] [33] [30] [32] [34]
Anaphylaxis to insect venom/food/drugs/idiopathic	***TPSAB1***	Tryptase alpha/beta 1	NM_003294.4: Increased a-tryptase copy	chr16:1240705-1242554		[30] [32] [34] [36] [37] [38] [39] [40] [42] [41]
Anaphylaxis to food/drugs	***STAT6***	Signal transducer and activator of transcription 6	NM_003153.5:c.1129G>A, p.(Glu377Lys)	chr12:57104547C>T		[48]
			NM_003153.5:c.1255G>C, p.(Asp419His)	chr12:57102879C>G		[49] [51]
			9 different rare variants			[50]
Anaphylaxis to food	***STAT3***	Signal transducer and activator of transcription 3	NM_139276.3:c.1699A>G, p.(Asn567Asp)	chr17:42323309T>C		[52]
Anaphylaxis to food, FDEIA	***FLG***	Filaggrin	NM_002016.2:c.441_442del, p.(Gly149GlufsTer4)	chr1:152314444delCT		[54]
			NM_002016.2:c.5368C>T, p.(Gln1790Ter); rs200622741	chr1:152309518G>A	p.Gln1790Ter	
			NM_002016.2:c.1501C>T, p.(Arg501Ter); rs61816761	chr1:152313385G>A	R501X	[55]
			NM_002016.2:c.2282_2285del, p.(Ser761CysfsTer36); rs558269137	chr1:152312601delACTG	2282del4	
Anaphylaxis to food, FDEIA	***NLRP3***	NLR family pyrin domain containing 3	NM_004895.5:c.2499-202T>C, p.?; rs4612666	chr1:247435768T>C		[56]
			NM_004895.5: c.*230G>C, p.?; rs10754558	chr1:247448734G>C		
WDEIA	***IL4***	Interleukin 4	Upstream Transcript Variant (rs2243250)	chr5:132673462C>T	IL-4-C590T	[57]
Anaphylaxis to food	***IL10***	Interleukin 10	NM_153758.5:c.−149 + 2474T>C, p.?; rs1800896	chr1:206773552T>C	IL10-1082G/A	[58]
WDEIA	***IL18***	Interleukin 18	Upstream Transcript Variant (rs1946518)	chr11:112164735T>G		[59]
Anaphylaxis to drugs	***HP***	Haptoglobin	NM_005143.5: del?	chr16:(?_72054504)_(?_72076977)del	Hp(del)	[64]
Anaphylaxis to insect venom/food/drugs	***AGT***	Angiotensinogen	NM_001384479.1:c.776T>C, p.(Met259Thr); rs699	chr1:230710048A>G	M235T	[61] [62]
Anaphylaxis to insect venom/food/drugs	***ACE***	Angiotensin I converting enzyme	NM_000789.3:c.2306-117_2306-116insAF118569.1:g.14094_14382, p.?;	chr17:63,488,531-63488532insAF118569:g.14094_14382	increased"I"genes	[60]
Anaphylaxis to drugs	***NAT2***	N-acetyltransferase 2	NM_000015.3:c.341T>C, p.(Ile114Thr), rs1801280	chr8:18400344T>C	NAT2*5	[63]
			NM_000015.3:c.[590G>A];[803G>A]	chr8:[18400593G>A];[18400806G>A]	NAT2*6	
			NM_000015.3:c.[803G>A];[857G>A]	chr8:[18400806G>A];[18400860G>A]	NAT2*7	
			NM_000015.3:c.[191G>A];[803G>A]	chr8:[18400194G>A];[18400806G>A]	NAT2*14	
Anaphylaxis to drugs	***CEP68***	Centrosomal protein 68	NM_015147.3:c.−46-4987A>G, p.?; rs2241160	chr2:65064412A>G		[65]
			NM_015147.3:c.−46-5691C>A, p.?; rs2241161	chr2:65063708C>A		
Anaphylaxis to insect venom/food/drugs	***CMA1***	Chymase 1	Upstream Transcript Variant (rs1800875)	chr14:24510132C>T	CMA-1A1903G	[62]

*Provided as transcript change, amino acid sequence change and dbSNP, if available, with the exception of HLA alleles ^♦^Omics studies

Over the past two decades, a great interest has been devoted to metagenomics, i.e., the analysis of all genomes of the microbiota (metagenome). Microbiota comprises the diverse community of microorganisms inhabiting a specific body site, including bacteria, archaea, viruses, fungi, and protozoans [8]. Two identified metagenomic studies on anaphylaxis suggest a potential role of microbiota in disease susceptibility [66, 67]. The first study investigated gut microbiota in patients with WDEIA, finding increased levels of *Blautia*, *Erysipelatoclostridium*, *Akkermansia*, and *Lachnospiraceae*, while *Lactobacillus* and *Dialister* were reduced compared to healthy controls. No significant differences in microbial diversity were observed, but specific bacterial taxa were correlated with IgE levels [66]. The second study focused on circulating microbiota in perioperative anaphylaxis patients and identified differences in the relative abundance of specific bacterial taxa, including *Enterobacteriaceae*, *Veillonellaceae*, *Escherichia*–*Shigella*, *Pseudarcicella*, *Rhodoferax, Saprospiraceae*, and *Lewinella* when compared to healthy controls. Furthermore, certain bacterial taxa were associated with mast cell tryptase concentrations and specific IgE levels [67]. Both studies suggest that microbiota composition, rather than overall diversity, may play a role in the pathophysiology of anaphylaxis (Table 2) [66, 67].

**Table 2:** Metagenomic studies on anaphylaxis

Pathology	Sample	Species diversity	Reference
WDEIA	Fecal samples	***Blautia, Erysipelatoclostridium, Akkermansia, Lachnospiraceae_NK4A136_group* ↑* Lactobacillus, Dialister ↓***	[66]^♦^
Anaphylaxis to drugs	Serum samples	***Enterobacteriaceae, Veillonellaceae, Escherichia Shigella, Pseudarcicella, Rhodoferax* ↑ *Saprospiraceae, Lewinella ↓***	[67]^♦^

^♦^Omics studies.

### Epigenomics

Epigenomics is a field of omics science that examines the epigenetic profile of a cell, known as the epigenome. Epigenetic regulation refers to the reversible alterations in gene expression that do not involve changes in the DNA sequence. DNA methylation and histone modifications are among the most extensively characterized epigenetic mechanisms [8]. Two studies investigating epigenetics in anaphylaxis have been identified [68, 69]. One study aimed to explore the role of epigenetics, particularly methylation, in anaphylaxis severity by analyzing CpG sites in CD4 + lymphocytes. It revealed 203 CpG sites with differential DNA methylation associated with anaphylaxis severity. The analysis identified four interconnected CpG-gene groups associated with immune response, chemotaxis, and macroautophagy, involving genes related to neutrophil and macrophage activity. Additionally, novel causal links were discovered between methylation signatures of *PHACTR1* (cg06769918) and *ZNF121* (cg12084124) and reaction severity, with baseline methylation inversely influencing gene expression and reaction severity [68]. The second study focused on DNA methylation signatures in β-lactam-induced fatal anaphylactic shock. It included a small cohort of eight cases and six controls to identify potential forensic biomarkers and explore underlying molecular mechanisms. Researchers identified 1459 differentially methylated regions associated with β-lactam-induced anaphylaxis, primarily involving the MAPK signalling pathway. Additionally, 18 DNA methylation signatures were found to distinguish fatal anaphylactic shock cases from healthy individuals, highlighting the role of hypomethylation in regulating gene transcription and contributing to the severity of drug-induced anaphylaxis (Table 3) [69].

**Table 3:** Differentially methylated genetic regions and associated genes

Pathology	Differentially methylated positions	Affected genes	Gene name	Reference
Anaphylaxis to food	**cg06769918**	***PHACTR1***	phosphatase and actin regulator 1	[68]^♦^
	**cg12084124**	***ZNF121***	zinc finger protein 121	
Anaphylaxis to drugs	**cg10413550**	***SEC16A***	SEC16 homolog A, endoplasmic reticulum export factor	[69]^♦^
	**cg07120806**	***WHSC1***	nuclear receptor binding SET domain protein 2	
	**cg25481680**	***PCGF6***	polycomb group ring finger 6	
	**cg01008854**	***HVCN1***	hydrogen voltage gated channel 1	
	**cg16692004**	***TAB2***	TGF-beta activated kinase 1 (MAP3K7) binding protein 2	
	**cg22617643**	***RPL7***	ribosomal protein L7	

^♦^Omics studies.

### Transcriptomics

The transcriptome encompasses all RNA molecules, including protein-coding RNAs and non-coding RNAs (ncRNAs), produced by genome transcription under specific conditions in a particular tissue or cell [8]. NcRNAs, which do not encode proteins longer than 100 amino acids, are categorized into two main groups: small noncoding RNAs (sncRNAs), such as microRNAs and snoRNAs, and long non-coding RNAs (lncRNAs) [8]. In anaphylaxis research, peripheral blood is the primary source for transcriptomic profiling [68, 70–82]. Transcriptomic analyses have been conducted in patients experiencing anaphylaxis, both during and after the reaction. Our review identified four studies that examined the transcriptome after the resolution of anaphylactic episodes, providing insights into post-reaction gene expression patterns [70, 75, 77, 78]. One study identified eight differentially expressed genes (DEG) between patients with a history of HVA and healthy controls (*CFAP184*, *DIRAS3*, *DYRK1A*, *FGFRL1*, *HS.146933*, *KLF10*, *LOC145820*, *NMB*, *OR5AR1*, *TSPYL2*). After venom immunotherapy, only *DYRK1A* and *TSPYL2* showed significant changes, suggesting a shift toward an expression profile resembling that of healthy controls [70]. Another study found that in mastocytosis patients, gene expression profiles are different between patients with a history of HVA and those without. These findings suggest that the differentiation state of mast cells may be a critical determinant of sensitivity to anaphylaxis. Gene expression profiling could be useful for predicting the risk of anaphylaxis in patients with mastocytosis; however, further studies in larger patient cohorts are needed to confirm these findings and develop a predictive tool suitable for clinical application [75]. Additionally, mastocytosis patients with food hypersensitivity showed higher *TRAF4* expression, while those with HVA had lower *B3GAT1* expression, suggesting other potential biomarkers for anaphylaxis risk assessment [78]. One study explored the differences between NSAID (nonsteroidal anti-inflammatory drugs)-dependent and NSAID-independent lipid transfer protein (LTP)-induced anaphylaxis (LTPA). The results revealed distinct gene expression profiles, including alterations in gastrointestinal epithelial renewal, immune responses, and adenosine metabolism, which could help explain the different inflammatory patterns and clinical responses in patients with LTPA and NSAID-LTPA [77].

Other studies have investigated transcriptomic changes during anaphylaxis [68, 71–74, 76, 79–82]. Peripheral blood leukocyte activation plays a key role in the systemic immune response, as demonstrated by dynamic gene expression changes during acute reactions [74, 76, 80]. In a study of six anaphylaxis patients, gene expression analysis revealed minimal differences at emergency department arrival, but 67 genes were altered after one hour and 2,801 after three hours. Network analysis identified three upregulated inflammatory modules enriched for LPS-like and TNF activation signatures, with central hub genes regulating innate immune responses. These findings highlight the involvement of innate immune pathways in anaphylaxis [76]. A subsequent study validated these findings by demonstrating increased expression and protein levels of key immune mediators, particularly those associated with neutrophil activation (*S100A8*, *S100A9*, *TLR4*, *TREM1, MMP9, OSM*) and inflammatory signaling [80]. Whole blood bulk RNA-sequencing of patients with acute anaphylaxis yielded similar conclusions, showing upregulation of neutrophil signature [68, 82] while also identifying downregulation of blood basophil signature [82]. The important and specific role of basophils in anaphylaxis was supported by two previous studies, which reported lower whole-blood gene expression of *FCER1A*, *CPA3*, and *HDC* during anaphylaxis, correlating with a decreased absolute number of circulating basophils, thus confirming basophil migration and/or activation [79, 81]. Another transcriptomic study revealed dysregulated platelet aggregation and activation (downregulated *GATA1*, *TLN1*, *GP1BA*, *MPL*, *F13A1*, *SPARC*) in the early stages of severe anaphylactic reactions, which may be associated with the severity of the reaction [73]. The same research group also demonstrated the unique involvement of snoRNAs (upregulated *SNORD61*, *SNORD8*, *SNORD69*, *SNORD119*, *SCARNA21*, *SNORD110*, *SNORD59A*) in the pathogenesis of anaphylaxis, suggesting that these are not a general feature of systemic inflammation (Table 4) [72]. Only one single-cell RNA sequencing (scRNA-seq) study investigated immune responses in anaphylaxis [71]. Single-cell sequencing enables detailed analysis of individual cell types, providing a more precise and comprehensive understanding of cellular heterogeneity and specific immune responses [8]. The study, which included only two HVA patients, revealed an increased number of monocytes and identified innate immune responses, leukocyte activation, and cellular detoxification as central to the acute phase of anaphylaxis [71].

**Table 4:** Differentially expressed genes associated with anaphylaxis

Pathology	Gene symbol	Gene name	State*	Reference
Anaphylaxis to insect venom	***CFAP184, DIRAS3,FGFRL1, KLF10, NMB, OR5AR1***	Cilia and flagella associated protein 184, DIRAS family GTPase 3, dual specificity tyrosine phosphorylation regulated kinase 1 A, fibroblast growth factor receptor like 1, KLF transcription factor 10, neuromedin B, olfactory receptor family 5 subfamily AR member 1, TSPY like 2	Basal	[70]^♦^
Anaphylaxis to insect venom	***RBMY1A3P, DVL1, G0S2, LOC283487, CTTNBP2, SIGLEC6***	RNA binding motif protein Y-linked family 1 member A3 pseudogene, dishevelled segment polarity protein 1, G0/G1 switch 2, cortactin binding protein 2, sialic acid binding Ig like lectin 6	Basal	[75]^♦^
Anaphylaxis to insect venom	***B3GAT1***	TNF receptor associated factor 4, beta-1,3-glucuronyltransferase 1	Basal	[78]
Anaphylaxis to food/NSAID	***ENTPD2, HRH4, IL4R, IFNLR1, IL1RL1, CD8A, GATA2, NCR1, CD177***	Ectonucleoside triphosphate diphosphohydrolase 2, histamine receptor H4, interleukin 4 receptor, interferon lambda receptor 1, interleukin 1 receptor like 1, CD8 subunit alpha, GATA binding protein 2, natural cytotoxicity triggering receptor 1, CD177 molecule,	Basal	[77]^♦^
Anaphylaxis to insect venom/food/drugs/idiopathic	***IFITM1, HLA-DQA1, MMP9, OSM***	Interferon induced transmembrane protein 1, major histocompatibility complex, class II, DQ alpha 1, metallopeptidase 9, oncostatin M	Acute	[76]^♦^
Anaphylaxis to insect venom/food/drugs/idiopathic	***IL10, S100A9, MMP9, TREM1, OSM, S100A8, TLR4, CD64, FASL***	Interleukin 10, S100 calcium binding protein A9, matrix metallopeptidase 9, triggering receptor expressed on myeloid cells 1, oncostatin M, S100 calcium binding protein A8, toll like receptor 4, Fas ligand	Acute	[80]
Anaphylaxis to insect venom/food/drugs/idiopathic	***IL1R2, FOS, MMP9, DUSP1, CLEC4D***	Interleukin 1 receptor type 2; Fos proto-oncogene, AP-1 transcription factor subunit; matrix metallopeptidase 9; dual specificity phosphatase 1; C-type lectin domain family 4 member D	Acute	[74]^♦^
Anaphylaxis to food	***SAP30, NFKBIA, STK16, PFKFB2, CLEC4E, ARG1, UPP1, ECHDC3, RNF144B***	Sin3A associated protein 30, NFKB inhibitor alpha, serine/threonine kinase 16, 6-phosphofructo-2-kinase/fructose-2,6-biphosphatase 2, C-type lectin domain family 4 member E, arginase 1, uridine phosphorylase 1, enoyl-CoA hydratase domain containing 3, ring finger protein 144B	Acute	[68]^♦^
Anaphylaxis to insect venom/food	***ARG1, BMX, CCR3, CD177, CDK5R1, CLEC4D, CLEC4E, CPA3, CST7, CYSTM1, DUSP1, ECHDC3, FOS, GATA2, HDC, HRH4, IL18R1, IL18RAP, IL1R2, IL5RA, IRS2, KLHL2, MMP9, MYCL, ORM1, OSM, PADI4, PER1, PPP1R3D, PTGDR2, SAMSN1, SEMA7A***	Arginase 1, BMX non-receptor tyrosine kinase, C–C motif chemokine receptor 3, CD177 molecule, cyclin dependent kinase 5 regulatory subunit 1, C-type lectin domain family 4 member D, C-type lectin domain family 4 member E, carboxypeptidase A3, cystatin F, cysteine rich transmembrane module containing 1, dual specificity phosphatase 1, enoyl-CoA hydratase domain containing 3, Fos proto-oncogene, AP-1 transcription factor subunit, GATA binding protein 2, histidine decarboxylase, histamine receptor H4, interleukin 18 receptor 1, interleukin 18 receptor accessory protein, interleukin 1 receptor type 2, interleukin 5 receptor subunit alpha, insulin receptor substrate 2, kelch like family member 2, matrix metallopeptidase 9, MYCL proto-oncogene, bHLH transcription factor, orosomucoid 1, oncostatin M, peptidyl arginine deiminase 4, period circadian regulator 1, protein phosphatase 1 regulatory subunit 3D, prostaglandin D2 receptor 2, SAM domain, SH3 domain and nuclear localization signals 1, semaphorin 7A	Acute	[82]^♦^
Anaphylaxis to insect venom/food	***FCER1A, CPA3, HDC***	Fc epsilon receptor Ia, carboxypeptidase A3, histidine decarboxylase	Acute	[79]
Anaphylaxis to insect venom/food/drugs/idiopathic	***FCER1A, CPA3, HDC***	Fc epsilon receptor Ia, carboxypeptidase A3, histidine decarboxylase	Acute	[81]
Anaphylaxis to insect venom/food/drugs	***GATA1, TLN1, GP1BA, SELP, MPL, F13A1, SPARC***	GATA binding protein 1; talin 1; glycoprotein Ib platelet subunit alpha, selectin P; MPL proto-oncogene, thrombopoietin receptor; coagulation factor XIII A chain, secreted protein acidic and cysteine rich,	Acute	[73]^♦^
Anaphylaxis to insect venom	***CLU, PLAC8, CES1, FTL***	Clusterin, placenta associated 8, carboxylesterase 1, ferritin light chain	Acute	[71]^♦^
Anaphylaxis to drugs	***SEC16A, NSD2, PCGF6, TAB2, HVCN1, RPL7***	SEC16 homolog A, endoplasmic reticulum export factor; nuclear receptor binding SET domain protein 2; polycomb group ring finger 6; TGF-beta activated kinase 1 (MAP3K7) binding protein 2; hydrogen voltage gated channel 1;ribosomal protein L7	Postmortem	[69]

Underlined genes show differential expression in at least two studies.

*Measured either during anaphylaxis (Acute state), after recovery (Basal state) or postmortem.

^♦^Omics studies.

Four studies have reported that miRNAs contribute to the pathogenesis of anaphylaxis [83–86]. An increase in miR-21-3p and miR-487b-3p levels during the acute phase of anaphylaxis was observed in studies involving children with food-induced reactions [83, 86]. Moreover, elevated values of miR-451a have also been described in 16 adults with food- and HVA [84]. Finally, miRNA levels have been characterized in adults with drug-mediated reactions and found a decrease of miR-375-3p levels that correlated with the increase in inflammatory cytokines and had a negative role in endothelial barrier stability [85]. One study characterized the serum profile of other sncRNAs beyond miRNAs in two groups: five adults with drug-induced anaphylaxis and five children with food-mediated anaphylaxis [87]. The study identified distinct sncRNA profiles in each group, with 33 and 80 differentially expressed sncRNAs, respectively. Notably, only three (Y_RNA.394, Y_RNA.781, and SCARNA2) were common to both groups, highlighting their potential as biomarkers and novel mediators of anaphylaxis (Table 5) [87].

**Table 5:** Small non-coding RNAs (sncRNAs) in anaphylaxis

Pathology	sncRNAs changes	State*	Reference
Anaphylaxis to food	**miR-21-3p ↑**	Acute	[83]^♦^[86]
	**miR-487b-3p ↑**	Acute	[83]^♦^
Anaphylaxis to insect venom/food/drugs/idiopathic	**miR-451a ↑**	Acute	[84]^♦^
Anaphylaxis to drugs/food	**miR-375-3p ↓**	Acute	[85]^♦^
Anaphylaxis to drugs/food	**Y_RNA.394 (food) ↓(drugs) ↑**	Acute	[87]^♦^
	**Y_RNA.781 (food) ↓(drugs) ↑**	Acute	
	**SCARNA2 ↓**	Acute	
Anaphylaxis to insect venom/food/drugs/idiopathic	**SNORD61, SNORD8, SNORD69, SNORD119, SCARNA21, SNORD110, SNORD59A ↑**	Acute	[72]^♦^

*Measured either during anaphylaxis (Acute state), after recovery (Basal state) or postmortem

^♦^Omics studies

### Proteomics

Proteomics is dedicated to the comprehensive characterization of proteins, offering insights into their structure, function, and interactions. Proteome is defined as the complete set of proteins in an organism, organ, tissue, or cell [17]. Our screening found no large-scale proteomic studies on anaphylaxis, with existing research primarily focusing on selected proteins. The proteins most extensively studied in anaphylaxis are those associated with mast cells, the primary effector cells driving the allergic response. Among these, tryptase is particularly significant due to its specificity for mast cell degranulation and utility as a clinical biomarker for confirming anaphylaxis diagnosis. Several studies have documented elevated serum and tissue tryptase levels during anaphylaxis [79, 81, 84, 88–93], with increased acute serum tryptase levels frequently serving as a diagnostic marker for mast cell activation and anaphylaxis. Higher baseline tryptase levels are associated with more severe HVA [94–97]. As a mast cell-associated receptor involved in degranulation, MRGPRX2 was investigated for its potential as a biomarker for predicting anaphylaxis. Findings indicated that serum MRGPRX2 levels were significantly higher in patients with iodinated contrast media-induced anaphylaxis compared to non-anaphylaxis cases [98]. Carboxypeptidase A is a zinc-dependent metalloprotein present as a preformed mediator in mast cells. Elevated levels of this protein have been detected in the serum of patients experiencing anaphylaxis and in those who have died from drug-induced reactions [90]. Additionally, other mediators derived from mast cells and basophils, including cytokines and chemokines, have been found to be elevated during anaphylaxis. Tumor necrosis factor α (TNF-α) is among the most extensively studied cytokines, with increased serum levels reported in patients experiencing anaphylaxis [92]. Furthermore, elevated concentrations of its receptor, TNF receptor I (TNFRI), have been observed in the majority of severe anaphylaxis cases [92]. Chemokines contribute to the recruitment and migration of various leukocyte populations during immune responses. Notably, C–C motif chemokine ligand 2 (CCL2) has been found to be elevated in samples from patients experiencing anaphylaxis [79, 81, 99]. The term"interleukin"(IL) was originally introduced to describe cytokines produced by leukocytes that function as messengers within the immune system [100]. However, these mediators can also be synthesized by various other cell types. Elevated serum levels of IL-2, IL-4, IL-5, IL-6, IL-10, and IL-13 have been reported in patients with severe anaphylaxis. Notably, IL-2, IL-6, and IL-10 correlate with the severity of anaphylactic episodes [80, 92, 99]. The complement system consists of a complex cascade of proteases that play a crucial role in both innate and adaptive immune responses. Elevated levels of the anaphylatoxins C3a, C4a, and C5a, which bind to specific G protein–coupled receptors on mast cells and basophils, have been detected in the sera of patients experiencing anaphylaxis and seem to correlate with the severity of the reaction [101].

Oher circulating proteins have also been characterized in anaphylaxis. Apolipoprotein B has been shown to inversely correlate with anaphylaxis severity [102, 103]. Additionally, a reduction in serum apolipoprotein A1 has been proposed as a promising biomarker for this pathological event [84, 103, 104]. A decrease in apolipoprotein E levels has also been observed in the serum of anaphylactic patients. However, since apolipoprotein E declines in other inflammatory conditions as well, it may not serve as a specific marker for anaphylaxis [104].

A recently published study investigated gastrointestinal permeability in patients with idiopathic anaphylaxis and found that I-FABP (intestinal fatty acid binding protein) and sCD14 (soluble CD14) were significantly elevated in patients compared to healthy controls, suggesting that increased gastrointestinal permeability may play a role in anaphylaxis pathogenesis [105]. Allergin-1, pregnancy-associated plasma protein-A, and galectin-3 were also identified to discriminate between patients with mastocytosis who experienced anaphylaxis and those who did not. These proteomic biomarkers may help predict which mastocytosis patients are at an increased risk of anaphylaxis (Table 6) [106].

**Table 6:** Proteins in anaphylaxis

Pathology	Protein name	State*	Reference
Anaphylaxis to insect venom/food/drugs/idiopathic	**Total tryptase (Tryptase alpha/beta-1, Tryptase beta-2) ↑**	Acute	[79] [81] [84] [88] [89] [93] [108] [109] [110]
Anaphylaxis to insect venom/food	**Total tryptase (Tryptase alpha/beta-1, Tryptase beta-2) ↑**	Basal	[94] [95] [96] [97]
Anaphylaxis to drugs	**Total tryptase (Tryptase alpha/beta-1, Tryptase beta-2) ↑**	Postmortem	[90, 91]
Anaphylaxis	**Mas-related G-protein coupled receptor member X2 ↑**	Basal	[98]
Anaphylaxis to drugs	**Carboxypeptidase A ↑**	Postmortem	[90]
Anaphylaxis to insect venom/food/drugs/idiopathic	**TNF receptor I ↑**	Acute	[92] [99] [101]
Anaphylaxis to insect venom/food/drugs/idiopathic	**C–C motif chemokine 2 ↑**	Acute	[81] [79] [99]
Anaphylaxis to insect venom/food/drugs/idiopathic	**Interleukin-2 ↑**	Acute	[92] [101]
Anaphylaxis to insect venom/food/drugs/idiopathic	**Interleukin-6 ↑**	Acute	[92] [101]
Anaphylaxis to insect venom/food/drugs/idiopathic	**Interleukin-10 ↑**	Acute	[92] [99] [101]
Anaphylaxis to insect venom/food/drugs/idiopathic	**Interleukin-4 ↑, Interleukin-5 ↑, Interleukin-13 ↑, Interferon gamma ↑, Tumor necrosis factor ↑**	Acute	[92]
Anaphylaxis to insect venom/food/drugs/idiopathic	**C3a ↑, C4a ↑, C5a ↑**	Acute	[101]
Anaphylaxis to insect venom/food/drugs/idiopathic	**Apolipoprotein A1 ↓**	Acute	[84] [103] [104]
Anaphylaxis to food	**Apolipoprotein B ↓**	Acute	[102] [103]
Anaphylaxis to insect venom/food/idiopathic	**Apolipoprotein E ↓**	Acute	[104]
Idiopathic anaphylaxis	**Intestinal fatty acid binding protein ↑, soluble CD14 ↑**	Basal	[105]
Anaphylaxis	**Allergin-1 ↓, Pregnancy-associated plasma protein-A ↓, Galectin-3 ↑**	Basal	[106]
Anaphylaxis to food/drugs	**Factor XII ↑, kallikrein ↑, kininogen ↑**	Acute	[111]
Anaphylaxis to food/drugs	**CDC42↑, Ficolin-2↑, S100A9↑**	Acute	[107]^♦^

*Measured either during anaphylaxis (Acute state), after recovery (Basal state) or postmortem

^♦^Omics studies

Extracellular vesicles (EVs) have emerged as key mediators of cellular communication and promising disease biomarkers. A proteomics-based study of EVs isolated from patients’ plasma during anaphylaxis identified 99 differentially expressed proteins, 83 of which were elevated during the acute phase, suggesting their potential as biomarkers. Among these, CDC42, Ficolin-2, and S100A9 were validated as elevated, linking them to immune signaling, inflammation, and endothelial activation. Pathway analysis revealed enrichment in processes such as leukocyte migration and cell degranulation [107].

### Metabolomics

Metabolomics, the comprehensive study of small-molecule metabolites in biological systems, has emerged as a promising approach for understanding the biochemical mechanisms underlying different diseases. Despite the growing interest in metabolomics, only one study investigating metabolic changes associated with anaphylaxis has been identified [112]. This study offers new insights into the metabolic alterations associated with anaphylaxis, demonstrating that different anaphylactic triggers or severity induce differential metabolic changes along time or at specific time points, respectively. Notably, these changes are observed in phospholipid metabolism, amino acid utilization, and energy metabolism [112]. Several metabolites linked to the activation of immune cells during anaphylaxis have been investigated individually. Histamine is a biogenic amine pre-stored in the granules of mast cells and basophils and is considered the primary mediator of anaphylactic reactions [113]. Studies have consistently reported elevated histamine concentrations during anaphylaxis [89, 92, 109, 114]. IgG-mediated anaphylaxis has been described in humans and may also be responsible for numerous cases of IgE-independent reactions. IgG antibodies act through binding to Fc gamma receptors (FcγRs), which are expressed in various cell types, including macrophages, dendritic cells, neutrophils, platelets, basophils, and mast cells. Neutrophils are believed to play a central role in IgG-mediated anaphylaxis [115]. One of the key molecules stored in neutrophils is platelet-activating factor (PAF), whose role in the pathogenesis of anaphylaxis has been suggested by several studies [108, 116]. Studies conducted by Vadas et al. demonstrated that PAF levels increased in correlation with the severity of anaphylactic reactions in both adult and pediatric patients [108, 110]. Additionally, PAF might be a more reliable diagnostic biomarker, with a higher percentage of patients identified based on PAF levels compared to other biomarkers such as histamine and tryptase [108]. The primary enzyme responsible for the degradation of PAF is PAF acetylhydrolase. This enzyme has been proposed as a potential molecular marker for anaphylaxis, with two studies indicating a decrease in its levels during anaphylactic reactions [116, 117]. Unfortunately, there is a paucity of additional studies to validate the role of PAF or PAF acetylhydrolase in the pathogenesis of anaphylaxis. Arachidonic acid metabolites, including cysteinyl leukotrienes (CysLTs) and prostaglandins (PGs) play important roles in the pathophysiology of various inflammatory diseases [118]. Urinary leukotriene E_4_ (LTE_4_) is considered the most reliable biomarker for assessing endogenous CysLTs synthesis, while urinary 9α,11β-PGF2 and the later appearing 2,3-dinor-9α,11β-PGF2, serve as relatively major metabolites of PGD2[118]. Several studies have reported increased levels of LTE_4_[118, 119] and 9α,11β-PGF2[84, 104, 118, 120] during anaphylaxis, further supporting their relevance as biomarkers for this condition.

The contact system, composed of proteases and coagulation factors, is activated by inflammatory macromolecules or negatively charged surfaces, leading to bradykinin production and intrinsic coagulation cascade activation. In anaphylaxis, heparin released from mast cells and basophils promotes factor XII autoactivation, triggering kallikrein-mediated bradykinin formation, a key vasoactive mediator in this reaction [121]. One study revealed activation of factor XII, kallikrein, and kininogen during the acute phase of anaphylaxis. The severity of anaphylaxis was also associated with mast cell degranulation, increased plasma heparin levels, the intensity of contact system activation, and bradykinin formation. These findings highlight the role of the contact system in anaphylaxis and support the hypothesis that targeting bradykinin generation and signaling could offer a novel therapeutic strategy for anaphylactic attacks (Table 7) [111].

**Table 7:** Metabolites and anaphylaxis

Pathology	Metabolites	State*	Reference
Anaphylaxis to food/drugs	**Cortisol, glucose, lipids (-CH = CH-CH2-CH = CH-), lipids (-CH = CH-), lipoprotein methyl group signal ↑**	Acute	[112]^♦^
	**Oleamide, PC(14:0/20:4), lactate, lipids (-CH2-CH = CH), lipids (-CH = CH-CH2-CH = CH-), lipids (-CH = CH-) ↑, betaine, cortisol *↓***	Basal	
Anaphylaxis to insect venom/food/drugs/idiopathic	**Histamine ↑**	Acute	[89] [92] [114] [109] [108]
Anaphylaxis to insect venom/food/drugs/idiopathic	**PAF ↑**	Acute	[116] [108]
Anaphylaxis to insect venom/food/drugs/idiopathic	**PAF acetylhydrolase ↓**	Acute	[116] [110]
Anaphylaxis to insect venom	**PAF acetylhydrolase ↓**	Basal	[117]
Anaphylaxis to food/drugs	**Leukotriene E4 ↑**	Acute	[118] [119]
Anaphylaxis to insect venom/food/drugs/idiopathic	**9α,11β-PGF2 ↑**	Acute	[84] [104] [118] [119] [120]
Anaphylaxis to insect venom/food	**PGE2 ↓**	Acute	[118] [120]
Anaphylaxis to food/drugs	**Heparin ↑**	Acute	[111]
Anaphylaxis to insect venom	**PGIM ↓**	Acute	[120]

*Measured either during anaphylaxis (Acute state), after recovery (Basal state) or postmortem

^♦^Omics studies

### Integrated Omics

While each omic layer provides valuable data on its own, their integration offers new and valuable insights. Combining multi-omics data can reveal novel cell subtypes, cell interactions, and cross-talk across different omic layers. Since each omic layer is causally connected to the next, multi-omics integration helps to unravel these complex relationships [122].

**Fig. 2:**
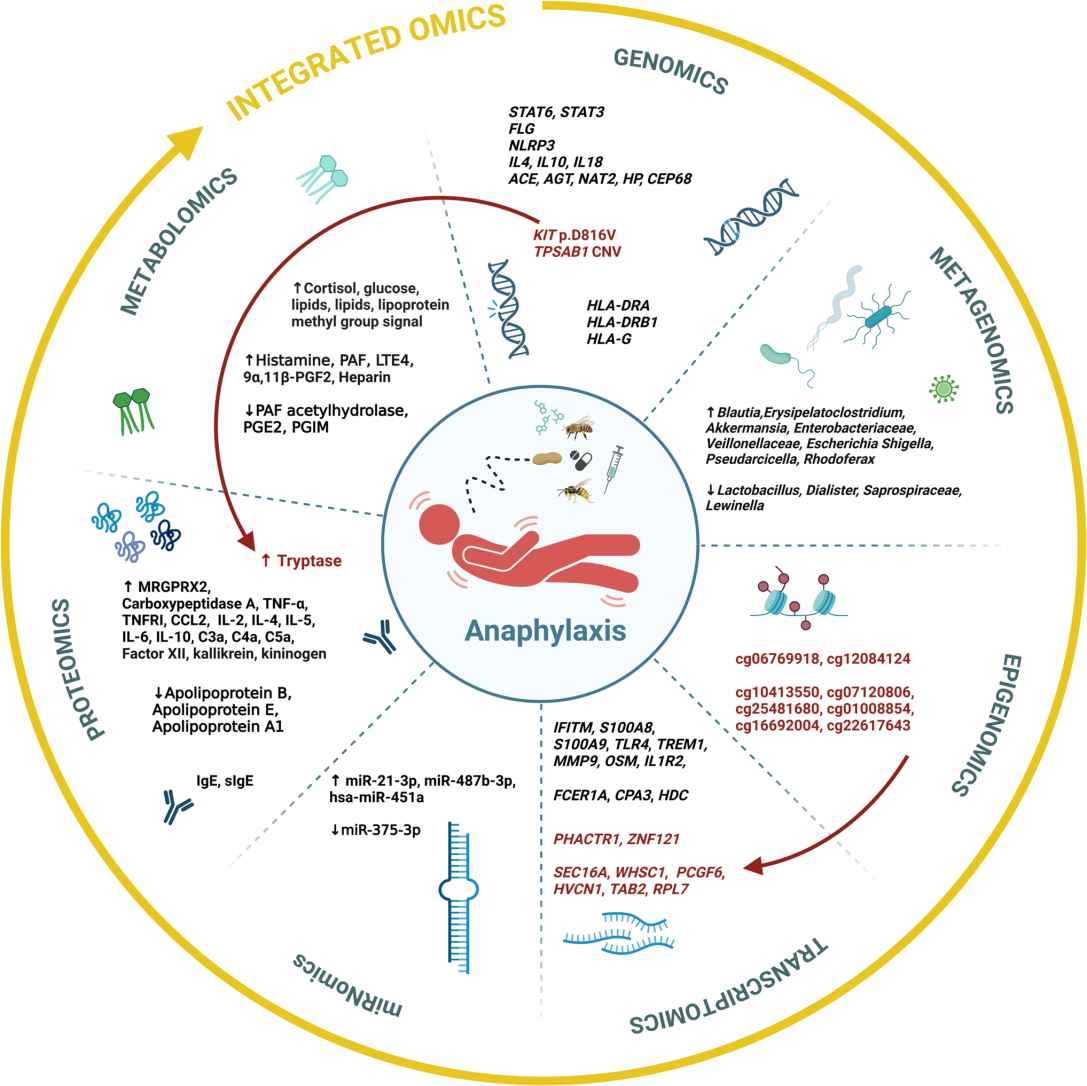
Findings across various omics levels and established associations between single-omics datasets in anaphylaxis. Red arrows indicate the key associations identified between the individual omics datasets

A PubMed database screening revealed only one study in the field of anaphylaxis utilizing an integrated omics approach [68]. This study aimed to understand better the mechanisms underlying the severity of peanut allergy reactions. By integrating transcriptomic and epigenomic data from peanut-allergic children during double-blind, placebo-controlled peanut challenges, the researchers identified genes and DNA methylation sites associated with reaction severity. Notably, methylation at specific CpG sites was found to influence the gene expression of *PHACTR1* and *ZNF121*, suggesting that DNA methylation may serve as an anchor for modulating reaction severity [68]. In Fig. 2, we present this multi-omics finding alongside other established associations between single-omics datasets identified in the available literature. This figure provides a comprehensive overview of omic profiles in anaphylaxis, integrating key known connections. Despite these insights, omics studies on anaphylaxis remain limited, highlighting the need for further research in this area.

## Conclusion and Future Directions

Anaphylaxis is a hypersensitivity reaction driven by complex molecular mechanisms that remain incompletely understood. Despite advancements in genomics, epigenomics, transcriptomics, proteomics, and metabolomics, the identification of reliable biomarkers and therapeutic targets remains a challenge.

Multi-omics technologies offer a promising approach to elucidating the complex pathophysiology of anaphylaxis, revealing critical genetic variants, immune regulatory pathways, and metabolic alterations associated with disease onset and severity. However, multi-omics data integration in anaphylaxis research remains limited, with only a few studies adopting comprehensive multi-layered approaches. The integration and normalization of data across omics remain challenging. Each omic layer exhibits distinct data structures and inherent variability, complicating effective integration and analysis. Future research should prioritize large-scale, multi-center investigations employing integrative multi-omics strategies, ideally using samples from the same patients and/or the same anaphylaxis trigger. HVA and food allergy may serve as ideal models for such research. HVA involves natural exposure to relatively consistent amounts of sting venom, making it an excellent model for assessing responsiveness to intravenous antigens in an emergency department setting. Furthermore, the severity of reactions tends to be repeatable, as a history of severe reactions to stings often correlates with subsequent severe events [123]. In the context of food allergy, a controlled oral food challenge serves as the most objective and quantitative method for determining the eliciting dose of the antigen and evaluating the severity of the reaction. When integrated into a comprehensive multi-omics framework, this approach could pave the way for precision medicine in anaphylaxis, enabling improved risk stratification, personalized treatments, and enhanced diagnostic accuracy. Moreover, standardized assay protocols and development of rapid, cost-effective diagnostic tools, together with professional training and clear regulatory and reimbursement pathways, will be key to translating research into routine care.

Despite promising discoveries, clinical translation of omics-based biomarkers in anaphylaxis faces several challenges. Key issues include limited validation due to small, heterogeneous cohorts and a lack of multicenter studies. High costs and technical demands of high-throughput omics platforms restrict routine use, while variability in sample handling and analysis further hampers standardisation. Anaphylaxis is an acute, rapidly evolving condition. Biomarker levels can fluctuate with timing during anaphylactic episodes, reducing consistency. Furthermore, the complex, multifactorial nature of anaphylaxis often requires multi-omic biomarker panels, which are difficult to validate and implement in clinical practice. These challenges collectively limit current feasibility and widespread clinical integration.

Nevertheless, some biomarkers are already used in clinical practice to support the diagnosis and management of anaphylaxis. Serum tryptase is the most widely used; a rise during the acute phase supports the diagnosis of anaphylaxis, although it is not elevated in all patients. Baseline tryptase levels are also assessed to evaluate the risk of severe reactions or underlying mast cell disorders, as elevated levels are associated with both. Moreover, the *KIT* p.D816V variant and HαT are relevant in the context of predisposition to severe anaphylaxis; their detection requires specialised equipment and expertise, which limits their availability to specialised referral centers and increases associated costs. Additionally, histamine and its metabolites were found to be elevated in plasma or urine shortly after reaction onset; however, their short half-life limits their practical use. PAF, despite being proposed as a promising biomarker for anaphylaxis severity, is difficult to measure accurately due to its rapid degradation by PAF-acetylhydrolase and inherent instability, hindering its routine use in clinical laboratories.

## Data Availability

No datasets were generated or analysed during the current study.
