# Correction: Towards a Multi-omics Understanding of Anaphylaxis: Insights into Pathogenesis and Biomarker Identification

**DOI:** 10.1007/s12016-025-09091-w

**Published:** 2025-08-07

**Authors:** Manca Svetina, Tanja Kunej, Peter Korošec, Matija Rijavec

**Affiliations:** 1 University Clinic of Respiratory and Allergic Diseases Golnik, Golnik, Slovenia; 2 Biotechnical Faculty, University of Ljubljana, Ljubljana, Slovenia; 3 Faculty of Pharmacy, University of Ljubljana, Ljubljana, Slovenia

**Corection: Clinical Reviews in Allergy & Immunology (2025) 68:61**

10.1007/s12016-025-09069-8

In this article, several words appear to have been inadvertently truncated in the first paragraph of the “Materials and methods” section. The phrases were incorrect displayed as ““chromati,” “DN,” “epigenetic,” “epigenom,” “epigenomic,” “exposom,” “exposomic,” “expressio,” “gen,” “genom,” “genomic,” “GWA,” “histon,” “lncRN,” “metabo¬lomic,” “metagenomic,” “methylatio,” “microarra,” “microbiom,” “miRN,” “mRN,” “mutatio,” “ncRN,” “omi,” “polymorphis,” “protei,” “proteom,” “proteomic,” “RN,” “SN,” “transcrip,” “transcriptomic,” “varian,” and “mediato.”” when it should have been ““chromatin,” “DNA,” “epigenetics,” “epigenome,” “epigenomics,” “exposome,” “exposomics,” “expression,” “gene,” “genome,” “genomics,” “GWAS,” “histone,” “lncRNA,” “metabolomics,” “metagenomics,” “methylation,” “microarray,” “microbiome,” “miRNA,” “mRNA,” “mutation,” “ncRNA,” “omic,” “polymorphism,” “protein,” “proteome,” “proteomics,” “RNA,” “SNP,” “transcript,” “transcriptomics,” “variant,” and “mediator.””

On the other hand, minor spacing and formatting errors were also identified in Tables 1, 2, and 3 which were also fixed in the updated online version of this article.

The original article has been corrected.

